# Sensory sensitivity and visual discomfort are not associated with altered gamma oscillations: A test of the excitation-inhibition hypothesis

**DOI:** 10.1162/IMAG.a.1136

**Published:** 2026-02-12

**Authors:** Petroc Sumner, Georgina Powell, Alice Price, Hannah Thomas, Lucie S. Reed, Gavin Perry, Craig Hedge, Krish D. Singh

**Affiliations:** Cardiff University Brain Research Imaging Centre, School of Psychology, Cardiff University, Cardiff, United Kingdom; School of Psychology, University of Birmingham, Edgbaston, Birmingham, United Kingdom; School of Psychology, Aston University, Birmingham, United Kingdom

**Keywords:** sensory processing disorder, sensory over-responsivity, dynamic causal modelling

## Abstract

Many people experience aversive hypersensitivity (discomfort/visual stress) to stimuli such as bright lights, striped patterns, strobing, motion, or complex visual scenes such as supermarkets. Such sensory hypersensitivity is often associated with one or more of a range of neurological, psychiatric, and neurodevelopmental conditions or neurodivergence. The cortical mechanisms of sensory hypersensitivity, and reasons why it occurs with such a range of conditions, remain unknown. For three decades, theories have focused on excitation/inhibition balance, where visual discomfort reflects over-excitation relative to inhibition. Visual gamma oscillations induced by viewing stripes are an accepted index of excitation/inhibition, and are successfully modeled by a cortical circuit. Visual gamma is, therefore, predicted to be altered in people with high visual discomfort. We tested this in two studies. The first used circular moving gratings to evoke visual gamma, alongside self-reported scales for sensory sensitivity and for discomfort induced by viewing images (N = 166). We found no correlation of subjective sensitivity or discomfort with gamma frequency or amplitude (all r<0.1), or with the modeled excitation/inhibition parameters. In study 2, we recruited two groups of participants with high and low sensitivity to visual stripes (N = 23,27), and induced gamma with gratings of four different spatial frequencies. We found no group differences in gamma frequency, amplitude or modeled parameters. We conclude that visual discomfort is not simply explained by higher excitation/inhibition ratio in the visual cortex, despite the dominance of this assumed explanation.

## 1 Introduction

Across the population many people experience aversive hypersensitivity (also known as visual stress or sensory discomfort) to sensory stimuli such as bright lights, striped patterns, loud sounds, or certain smells (Price et al., 2025b; Tyler et al., 2014). Prevalence estimates vary from 3% to >20% (depending on severity definition and sampling or assessment method, e.g. Carpenter et al., 2019; Powell, Derry-Sumner, Rajenderkumar, et al., 2020; Tyler et al., 2014), but serve to demonstrate that the aversive experience is relatively common. Such sensory hypersensitivity is a key feature of autism and has a well-known association with migraine (Haigh et al., 2012), but it can also occur alone or with other neurological, psychiatric, and neurodevelopmental conditions or areas of neurodivergence (e.g. Price et al., 2025b). This transdiagnostic presentation could imply a common mechanism of vulnerability important for understanding brain development trajectories implicated in neurodiversity, mental health, and multiple clinical conditions (Price et al., 2025b).

However, the neural mechanisms of sensory hypersensitivity remain unknown, except for relatively rare cases where photophobia or auditory hyperacusis have a peripheral origin (in the eye, ear, or related nerve fibers, Noseda et al., 2019). The majority of reported hypersensitivity is more general and appears to have a cortical origin (Bargary et al., 2015; Patterson Gentile & Aguirre, 2020; Huang et al., 2003; Orekhova et al., 2019; Pienkowski et al., 2014). Importantly, it is an experience generated by supra-threshold stimuli and does not generally correlate with detection ability in psychophysical tasks (He et al., 2023; Schulz & Stevenson, 2022; Ward, 2019). The most elaborated theories focus on excitation/inhibition balance in sensory cortices (Fisher et al., 2022; O’Hare et al., 2023; Puts et al., 2011; Ward, 2019; Wilkins, 1995; Yoshimoto et al., 2017).

The development of this class of theory has mostly focused on visual sensitivity and activity within the visual cortex. A visual system that is sensitive to important stimuli without being over-reactive is dependent on local excitation/inhibition (E/I) balance. It is thought that visual cortices are tuned for sparse efficient coding of natural scenes, and are vulnerable to over-excite with stimuli that deviate from natural scene properties, such as stripes, especially with power at around 1 to 5 cycles per degree; for some people this vulnerability is thought to be enhanced, resulting in over-reactivity and aversion (Penacchio et al., 2023; Ward, 2019; Wilkins, 1995).

Of particular theoretical interest has been hypersensitivity experienced to repeating stripes, such as occurring in some architecture, or lines of text on a page. It has recently been shown that aversion to such striped patterns is one of the four correlated factors of visual hypersensitivity (Price et al., 2025a). Such patterns strongly deviate from the statistical properties found in natural environments in terms of spatial frequencies and orientation structure (Fernandez & Wilkins, 2008; Juricevic et al., 2010; Yoshimoto et al., 2017) and evoke large metabolic and electrophysiological responses in the visual cortex (Huang et al., 2003, 2011; Le et al., 2017; Singh et al., 2000). Such cortical responses are sometimes reported to be larger still in those susceptible to discomfort, migraine, or epilepsy (Haigh et al., 2012; Huang et al., 2003; Perry et al., 2014; Tempesta et al., 2021). Computational models of orientation and spatial frequency coding in V1 and V2 also overload to stimuli with a preponderance of mid-frequency stripes in consistent orientations across a scene (Penacchio & Wilkins, 2015; Penacchio et al., 2021, 2023).

Despite the long association of individual differences in sensory sensitivity with the concept of excitation/inhibition imbalance in the sensory cortex, the direct evidence for this theory is limited (Bargary et al., 2015). Visual gamma oscillations appear to be a robust biological trait marker indexing excitation/inhibition balance in visual cortex (Muthukumaraswamy et al., 2010; Tan et al., 2016). The relationship between gamma and excitation/inhibition is long established in animal and *in vitro* studies (Bartos et al., 2007; Buzsáki & Wang, 2012). In humans, synchronized gamma also seems to reflect excitation/inhibition, as supported by converging evidence from pharmacological interventions (Campbell et al., 2014; Lozano-Soldevilla et al., 2014; Magazzini et al., 2016; Saxena et al., 2013) as well as correlations with GABA_A_ receptor density (Kujala et al., 2015). Visual gamma shows stable individual differences across repeat testing (Muthukumaraswamy et al., 2010), appears to be genetically linked (van Pelt et al., 2012), and develop through early childhood consistent with maturation of the excitation/inhibition balance (Rhodes et al., 2024). Gamma frequency shows repeatable decrease with age (Gaetz et al., 2012; Muthukumaraswamy et al., 2010; Orekhova et al., 2015; Robson et al., 2015).

Therefore, the leading neural theory of sensory sensitivity—that of excitation/inhibition balance—predicts alterations in visual gamma oscillations. Indeed, it has long been thought no coincidence that the stimuli that most strongly generate visual gamma—stripes of around three cycles per degree—are also stimuli that cause aversive experiences in those sensitive to visual discomfort (e.g. Adjamian et al., 2004).

Visual gamma has been used to assess excitation/inhibition in sensory sensitivity previously, and also in autism, in which hypersensitivity is a key feature, but the results are not straightforward to interpret. No difference in gamma amplitude has been reported between autistic and non-autistic participants (Orekhova et al., 2019; Seymour et al., 2019). However, when moving stimuli are used to induce gamma oscillations, gamma amplitude increases from slow to medium speeds, and then reduces again for faster speeds (Orekhova et al., 2018). This reduction for intense stimuli is interpreted as gamma suppression, and people with higher sensory sensitivity had less of a reduction—lower ‘suppression’ (Manyukhina et al., 2021; Orekhova et al., 2019). A similar small effect was also found for autistic participants (Orekhova et al., 2023). A different study found that for noisy motion stimuli, the increase in gamma with motion coherence had a steeper slope in autistic participants, possibly indicating a higher gain function (Peiker et al., 2015). However, these effects—stronger gamma amplitude for specific stimuli in participants expected to have more aversive sensory sensitivity—are rather different from the established excitation/inhibition theory, in which lower inhibition should weaken gamma amplitude, for all generating stimuli.

Dynamic causal modelling (DCM; Moran et al., 2009, 2013) of neural dynamics underlying the generation of the oscillation spectrum (including gamma oscillations and other frequency bands) can now be used to relate oscillatory differences to modelled differences in cortical circuitry (Buzsáki & Wang, 2012; Shaw et al., 2017). Specifically, higher gamma frequency is related to stronger self-inhibition of superficial pyramidal cells (gain control) in the model, while higher gamma amplitude is related to stronger inhibition from interneurons on superficial pyramidal cells. Other parameters can also be fit, including excitatory drive from deep pyramidal cells to inhibitory interneurons, and self-inhibition of those interneurons. This modelled approach has been applied in schizophrenia, in typical development, and in the menstrual cycle to help focus theories of inhibitory differences or change onto specific mechanisms (Rhodes et al., 2024; Shaw et al., 2020; Sumner et al., 2018).

Here, we test whether sensory sensitivity is associated with alterations in visual gamma oscillations, and use DCM modelling with the aim of probing specific excitation or inhibitory mechanisms in local cortical circuitry. We follow the visual gamma technique used for previous DCM modelling studies and the pharmacological validation studies outlined above. We first tested the association of general sensory hypersensitivity with visual gamma as part of the Welsh Advanced Neuroimaging Database (WAND) cohort study (McNabb et al., 2025). In a second study, we test for a more specific association between visual gamma and sensory aversion to striped patterns, the type of hypersensitivity most associated with models of cortical over-excitation. By recruiting groups with high or low pattern sensitivity, we ensured that one group of participants were experiencing sensory discomfort at the time of gamma recording, to provide the best chance of detecting the neural correlate of that experience.

## 2 Methods

### 2.1 Study 1

#### 2.1.1 Participants

Study 1 was designed and conducted as part of the Welsh Advanced Neuroimaging Database (WAND) in which 170 healthy volunteers aged between 18 and 63 (median 25) took part in up to 8 neuroimaging sessions plus additional questionnaires and cognitive testing. Three participants had missing or unreadable data due to errors in the acquisition process. One participant had excessively noisy data due to the presence of some unknown metal on their person (analysed N = 166). This sample size is sufficient to detect an effect size of r = 0.21 (α = .05, β = .2, two-tailed). For demographic details and exclusion criteria see McNabb et al. (2025). Data are available at: https://gin.g-node.org/CUBRIC/WAND. Code for the analysis pipeline is available at https://osf.io/yrusq. Ethical approval for both studies was granted by the School of Psychology Ethics committee, Cardiff University.

#### 2.1.2 Magnetoencephalography

**Fig. 1.**
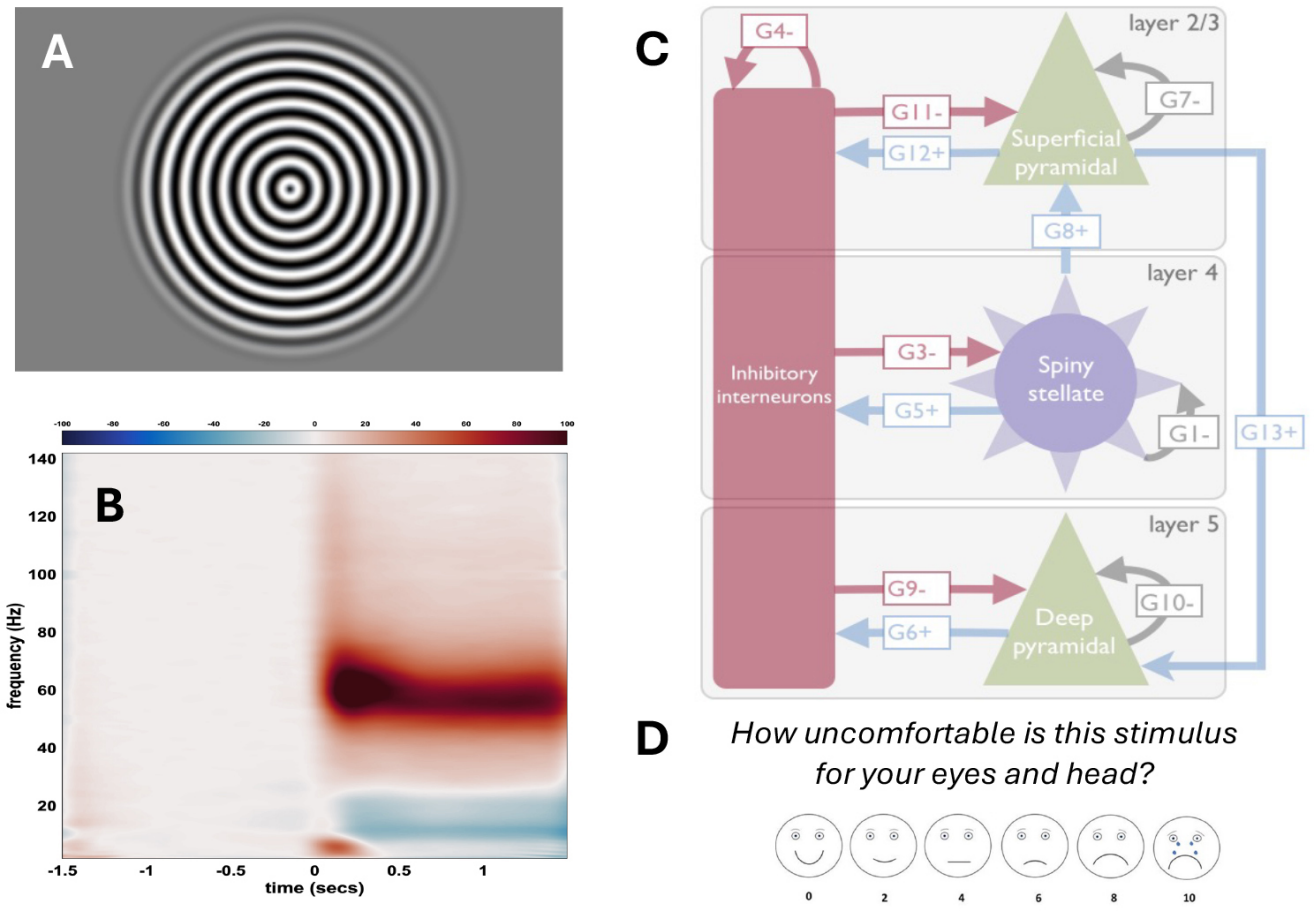
(A) Gamma oscillations in the visual cortex were induced by viewing high-contrast circular sinusoidal gratings. In study 1, these had spatial frequency 1.5 cpd and drifted inward. As an attention check, participants monitored the stimulus to detect a speed change. In Study 2, four spatial frequencies were used (0.75, 1.5, 3, and 6 cpd). (B) Time-frequency plot averaged across all participants in study 1, showing narrowband- induced gamma, was elicited as expected. (C) In order to provide insight into the neural populations generating stimulus-driven oscillations and how these may be modified in people with sensory sensitivity, we used dynamic causal modelling (DCM) of a canonical cortical circuit to fit the excitation and inhibition weights for each participant’s virtual sensor at the peak gamma location (illustration from Shaw et al., 2017). (D) In study 2, the participants’ task was to periodically rate their visual discomfort for each spatial frequency using a pictorial scale (trait visual hypersensitivity questionnaires were completed outside the scanner in both studies).

We presented 100 trials with stimuli known to induce visual gamma (Fig. 1A). For each trial, a white fixation dot was presented for 1.5 s; then, a circular sinusoidal grating with spatial frequency 1.5 cycles per degree (cpd) was presented (size 11.25 degrees; central 7.5 degrees at full contrast, then tapered toward zero following a raised cosine function) contracting toward the center at two-thirds of a degree per second (to aid fixation maintenance). While the peak gamma response is often reported to be around 3 cpd, the amplitude is fairly flat between 1 and 3 cpd (Adjamian et al., 2004 Fig. 2). After a random interval between 0.75 and 3 s, the stimulus speed doubled for 0.5 s and participants were instructed to indicate this by pressing a button with the right index finger (to ensure maintained attention). The grating was removed and feedback given for 1.5 s (“Too soon” if the button was pressed before the speed changed, “Too late” if the button was not pressed before the stimulus disappeared, or “OK”). Note that since gamma frequency increases sightly with speed, this stimulus change broadens the gamma peak. Stimuli were back-projected onto a screen inside the magnetically shielded room using a Propixx projector (Vpixx Technologies, Inc: Saint-Bruno, QC) running at a 1440 Hz refresh rate and a 960 × 540 resolution.

**Fig. 2.**
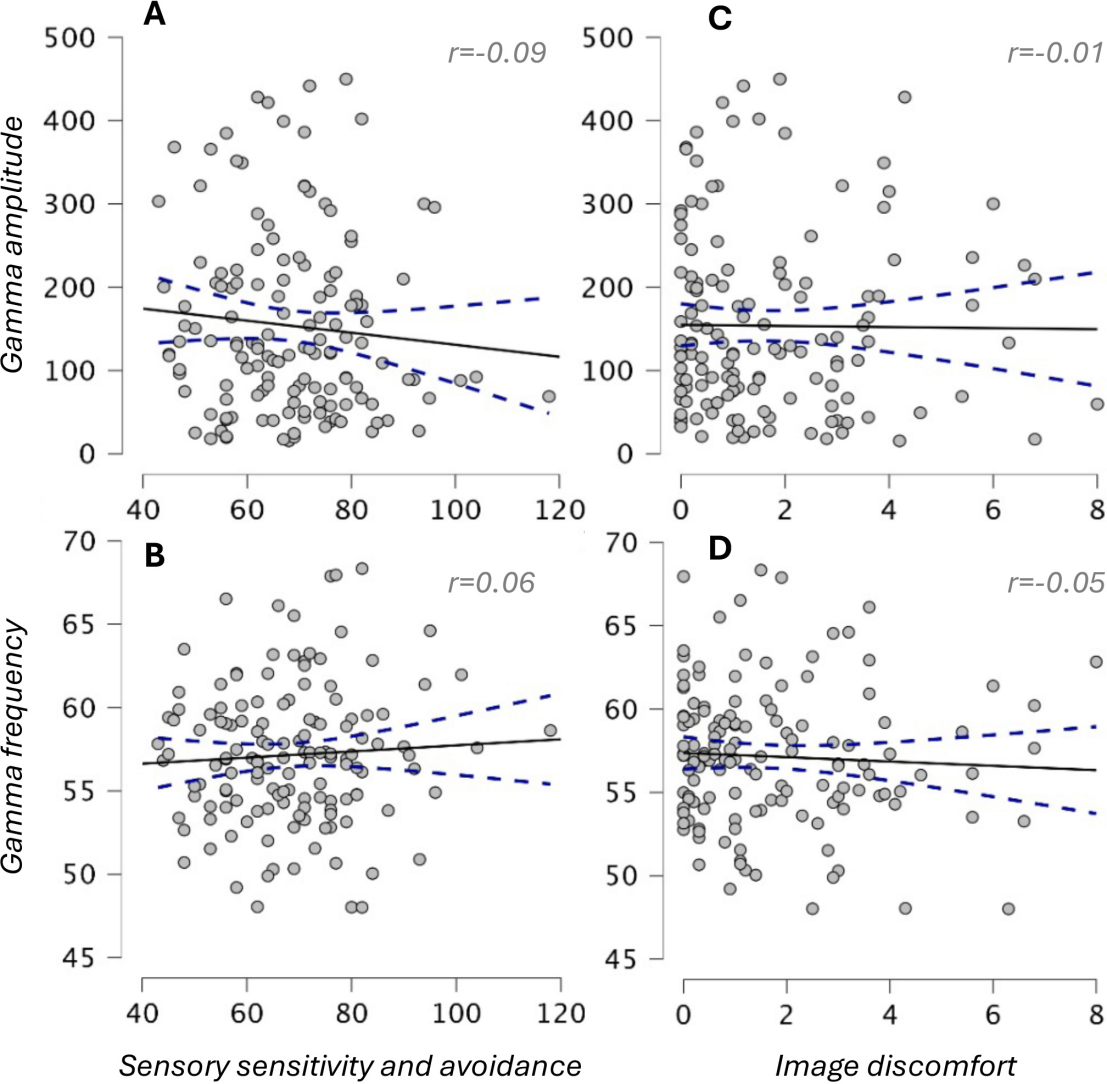
Neither gamma amplitude (A, C) or frequency (B, D) were predicted by sensory sensitivity and avoidance (A, B) or by image discomfort ratings (C, D) in Study 1. Axis labels at left refer to both plots in each row, and likewise labels at bottom refer to both plots in each column.

Whole-head MEG recordings were acquired at a 1200 Hz sampling rate on a 275-channel CTF radial gradiometer system. An additional 29 reference channels were recorded for noise cancellation purposes, and the primary sensors were analyzed as synthetic third-order gradiometers (Vrba & Robinson, 2001). Head digitization used a Polhemus digitizing system. MEG was conducted prior to any MRI, to avoid residual tissue magnetization. Participants sat upright with a chin rest to minimize movement. We also recorded electro-oculograms for blinks and eye movements, and bilateral wrist electrocardiogram. Time-frequency plots were checked to ensure narrowband gamma was induced as expected (Fig. 1B, for plot method see e.g. Swettenham et al., 2009).

T1-weighted anatomical MR scans were acquired for co-registration using an MPRAGE sequence with TR 2250 ms, TE 3.06 ms, flip angle 9°, FOV 256 × 288 × 176 mm^3^, voxel size 1 × 1 × 1 mm, TI 850 ms, 2-fold in-plane undersampling, and phase-encoding direction A>>P. To match MEG and MRI coordinate systems, each individual’s Polhemus head-shape was matched to scalp surface from their MRI using the FieldTrip toolbox.

#### 2.1.3 Beamforming

The MEG acquisition electronics apply a hardware anti-aliasing filter at ¼ of the sampling rate (300 Hz). No other filtering was applied prior to the beamformer analysis. After downsampling the MEG data to 300 Hz, source-localization was performed using the linear-constraint minimum-variance (LCMV) beamformer instantiated within FieldTrip (v20231113) (Oostenveld et al., 2011; Van Veen et al., 1997). MEG trials with artefacts (muscle, channel jumps), eye-movements, or head-movement > 5 mm from the median position were removed. A source model was constructed by segmenting the individual’s MRI into brain/non-brain tissues using SPM12’s ‘new’ segmentation algorithm. A conductivity head-model for the forward-problem was then constructed for this brain using FieldTrip’s singleshell option (Nolte, 2003). A 2 × 2 × 2 mm grid, defined in MNI template brain space, was then warped to this segmented brain to form the beamformer reconstruction grid. To reconstruct visual gamma, LCMV beamforming was performed in the posterior cortex (MNI range X:-5 to 5, Y: -12 to -6, Z: -7 to 6 cm) with a bandpass frequency range of 40 to 90 Hz (to avoid beta range influence) and a global covariance matrix. Virtual sensors were constructed for each grid position, and gamma amplitude was contrasted between the stimulus presentation period (300 to 1400 ms to encompass the steady-state gamma oscillation) and the baseline period (-1300 to -200 ms to avoid artefacts from adjacent trials). The virtual sensor at the peak response voxel was used for analyses of gamma amplitude and frequency. Power-spectra were calculated for each person using the multi-tapered FFT algorithm with spectral smoothing of ± 2Hz, implemented in FieldTrip’s *ft_freqanalysis* function. Separate spectra were derived from baseline and stimulus time-periods, converted to amplitude spectra via the *sqrt* function and stimulus-driven effects calculated as percentage-change from baseline. The peak-frequency was estimated as the centre-of-gravity of the response spectra in the 30–80 Hz range, based on our previous experience of individual gamma peaks (Robson et al., 2015; Shaw et al., 2017) and the amplitude as the mean amplitude over the same range in order to provide robust estimates (Lozano-Soldevilla et al., 2014).

#### 2.1.4 Neurophysiological modeling

We took the virtual sensors forward for analysis using the dynamic causal modelling (DCM) framework of SPM8 (https://www.fil.ion.ucl.ac.uk/spm/software/spm8/), as previously described (Shaw et al., 2017). In brief, we used the convolution-based canonical microcircuit model (CMC) shown in Figure 1C to generate model power spectra for the steady-state simulation period. The parameters of the model that affect the spectrum (G4-9, 11,12, see Shaw et al., 2017) are iteratively changed to best match the real virtual-sensor stimulus spectra for each participant, providing a participant-specific set of posterior model parameter estimates. As the parameters reflect either excitatory or inhibitory processes, then any participant-related differences in the E/I balance should be reflected in differences in one or more of these parameters. Of particular interest, following Shaw et al. (2017), are inhibitory gain control on the superficial pyramidal cells (G7 in Fig. 1C) and inhibitory drive from the inhibitory interneurons (G11). A full explanation of all parameters can be found in Shaw et al. (2017).

#### 2.1.5 Removal of weak gamma responses

For some participants there was no clear gamma response. To exclude these datasets in a principled way, we used the DCM model. The G7 parameter should be highly correlated with the peak gamma frequency (Shaw et al., 2017), and if apparent peaks with very low amplitude are real, they should align with this correlation. If they are noise, they would disrupt the correlation. Without any amplitude thresholding, this correlation was r = 0.59. Iteratively removing the weakest gamma participants produced a step-change to r = 0.77 when excluding 14 (of 166) participants; representing a gamma amplitude threshold of 14% change from pre-stimulus baseline (the cohort mean was 142%). Above this threshold, we conclude the gamma signal is meaningful for analysis. We, therefore, omitted participants with mean gamma amplitude <14%.

#### 2.1.6 Adolescent/adult sensory profile (AASP; Brown et al., 2001; Dunn, 1997)

The dimensions related to aversive sensory experience are *sensory sensitivity* (Cronbach alpha = 0.72) and *sensation avoiding* (Cronbach alpha = 0.77)*,* which tend to be highly correlated and were combined (summed) for our analysis, following Powell, Derry-Sumner, Shelton, et al. (2020) and Price et al. (2021). We also checked correlations with visual gamma using the visual questions from these scales alone (ignoring questions about other senses).

#### 2.1.7 Visual discomfort images

Participants rated (from 0 to 10) the extent of visual discomfort experienced when viewing 20 static images of abstract art, geometric shapes, and buildings previously shown to evoke discomfort for some people (Price et al., 2025). We used the mean rating across images (Cronbach alpha in the WAND cohort = 0.93).

#### 2.1.8 Data checks

We checked that expected levels of correlation were present with other measures in the WAND data known to correlate with sensory sensitivity (Powell et al., 2022; Price et al., 2021, 2025; Ward et al., 2017). These measures were: *Autism Quotient (AQ;* Hoekstra et al., 2011); *Hospital anxiety and depression scale (HADS;* Zigmond & Snaith, 1983)*; Visual vertigo analogue scale, (VVAS;* Dannenbaum et al., 2011)); *Migraine Screening Questionnaire (MS-Q*; Láinez et al., 2005).

### 2.2 Study 2

#### 2.2.1 Participants

We recruited 23 participants with high and 27 participants with low visual pattern discomfort, through screening with the pattern subscale of the Cardiff Hypersensitivity Scale (CHYPS), which assesses the four factors of visual discomfort (Price et al., 2025a, 2025b). Scores above the 75^th^ percentile on the subscale (4/15) were classified as high discomfort and zero or 1 as low discomfort. One participant had significant artefacts in their MEG recording due to presumed metal contamination and was excluded. Mean age (SD) of the final groups (High N = 22, Low N = 27) was 28 (12) and 26 (11) years. This sample size is sufficient to detect an effect size of d = 0.82 or r = 0.38 (α = .05, β = .2, two-tailed). Data and code to generate the virtual sensors, spectral reconstructions, and the peak gamma frequency and amplitude metrics are available at: https://osf.io/yrusq.

#### 2.2.2 Magnetoencephalography

The visual gamma protocol was identical to study 1 except as described below. We used four different spatial frequencies for the stimulus: 0.75, 1.5, 3, and 6 cpd (Fig. 1A) in order to also assess whether gamma power and discomfort ratings followed the same inverted U-shape across spatial frequency (Adjamian et al., 2004). Stimuli were static circular sinusoids presented for 1.5 s with random ISI between 2.25 and 2.5 s. There were 80 trials per condition (split into two runs). Beamforming and DCM model fitting was performed independently for each spatial frequency for each participant. The analyzed baseline and stimulus periods were -1500 to -300 ms and 300 to 1500 ms. All participants had at least one spatial frequency that yielded more than a 14% change from baseline, so no participants were removed using this threshold.

#### 2.2.3 Sensory discomfort measurement

On ten randomly selected trials per spatial frequency participants rated their visual discomfort in response to a 6-point scale of schematic faces (Fig. 1D), based on face scales used in pain research (Li et al., 2023; Tomlinson et al., 2010). To avoid confusing the participants, we only included one behavioral task, so there was not an additional attention check.

After the MEG session, participants completed the CHYPS again to confirm their high or low discomfort status, and a further set of brief questionnaires assessing known correlates of visual discomfort in order to sense-check the data as in study 1: anxiety (HADS-A), migraine (MS-Q), and visually-induced dizziness (VVAS) (AQ was omitted as it is a longer questionnaire).

### 2.3 Statistical approach

For study 1, we used Pearson correlation for planned correlations between sensory sensitivity measures and gamma amplitude or frequency. We followed up with correlations to check whether the key measures behaved as expected with respect to age and other measures in the collected battery. After fitting the DCM, we ran two linear regression models with DCM parameters as predictors and each sensory sensitivity measure as outcome. We used JASP with default priors for Bayesian analyses (JASP Team, 2025).

In study 2, we used ANOVA to assess group differences and interactions with spatial frequency. We used correlations to check expected behavior of measures, and regression for DCM parameters as in study 1.

## 3 Results

In Study 1, there were no significant correlations between sensory discomfort (AASP sensitivity/avoidance or discomfort image ratings) and visual gamma amplitude or frequency (Fig. 2). The correlations for gamma amplitude and frequency with sensory sensitivity/avoidance were, respectively, r = -0.09 (95% CI -.25 to +.08, Bayes Factor for null = 5.5) and r = 0.06 (95% CI -.11 to +.22, BFnull = 7.5). The correlations for gamma amplitude and frequency with discomfort image ratings were, respectively, r = -0.01 (95% CI -.18 to +.16, BFnull = 9.4) and r = -0.05 (95% CI -.22 to +.11, BFnull = 7.7).

**Fig. 3.**
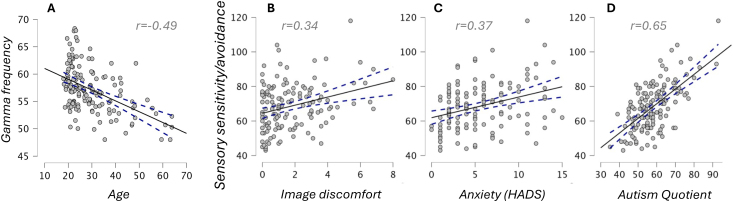
Sense checks for data quality in Study 1. (A) Gamma frequency is expected to decrease with age; sensory sensitivity and avoidance is expected to positively correlate with image discomfort ratings (B), anxiety scores, (C), and autism quotient, (D). Y-axis labels refer to all following plots.

As a sense check for the gamma data, the expected negative correlation between gamma frequency and age was strongly present (Fig. 3A; r = -0.49, 95% CI -.60 to -.36, p < 0.001). Note that correlation of frequency with sensory sensitivity measures remained absent when partialling out age (r = 0.05, r = -0.04; this was also true for amplitude: r =- 0.08, r = -0.01).

DCM parameters (Fig. 1C) were entered as regressors into a regression model with outcome sensory sensitivity/avoidance. This was repeated for outcome image discomfort, and then age (as a sense check). The DCM parameters did not predict either sensory sensitivity/avoidance (F = 0.3, BFnull>3 for all parameters) or visual discomfort ratings (F = 0.8, BFnull >3 for all parameters except G8, BFnull = 1.8). They did predict age as expected (F = 6.8, p < 0.001).

Sense-checks revealed no data quality issues with the questionnaires. The AASP sensitivity and avoidance scales correlated with each other as expected (r = 0.63, 95% CI .51 to .73, p < 0.001), and the combined scale correlated with the discomfort images (r = 0.34, 95% CI .18 to .49, p < 0.001), anxiety (r = 0.37, 95% CI .20 to .51, p < 0.001) and autism quotient (r = 0.65, 95% CI .54 to .74, p < 0.001) at values fully consistent with previous research (Fig. 3). The other expected correlations were also present: sensory sensitivity/avoidance or discomfort image ratings with the VVAS scale (r = 0.33, 95% CI .16 to .48, p < 0.001; r = 0.46, 95% CI .31 to .59, p < 0.001) and with migraine (r = 0.38, 95% CI .22 to .52, p < 0.001; r = 0.21, 95% CI .04 to .37, p < 0.05, note migraine had restricted variance in our data due to exclusion criteria in WAND). Since image discomfort scores are skewed, we also checked a log transform, which did not reveal correlations with gamma amplitude or frequency (r = -0.02, -0.08).

Exploratory analyses showed that restricting the AASP to only the visual questions did not make any difference to the absent correlations with gamma amplitude or frequency alone (r = -0.12, 95% CI -.28 to .05, BFnull = 3.5; r = 0.01, 95% CI -.16 to .17, BFnull = 9.5), and neither was visual AASP predicted by the DCM (F = 0.4, all BFnull>3). There was no effect of gender on any of the relationships of interest.

**Fig. 4.**
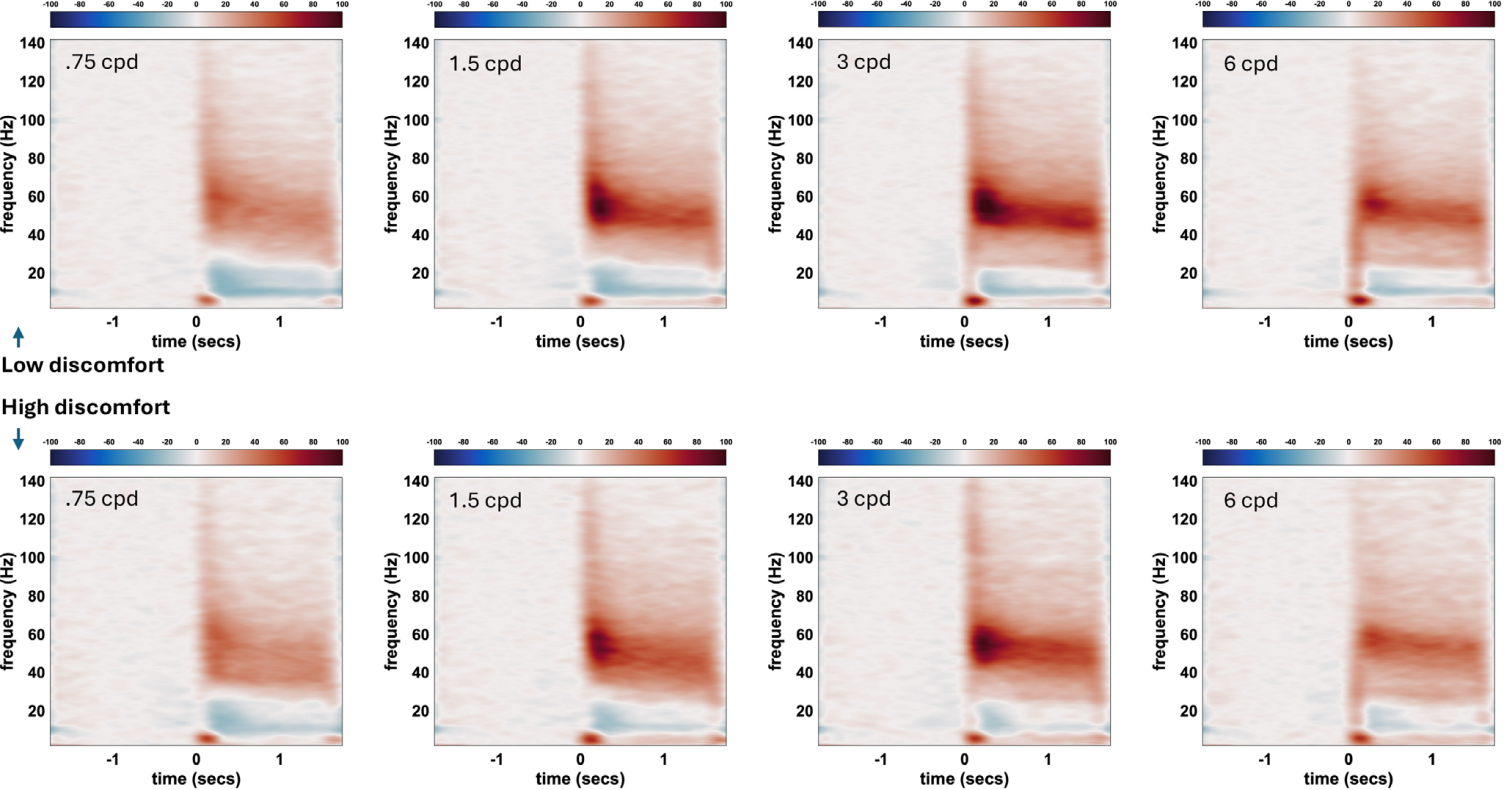
Time-frequency plots for each spatial frequency (left to right), for the low discomfort group (top row) and high discomfort group (bottom row). For plot methods see e.g. Swettenham et al. (2009).

**Fig. 5.**
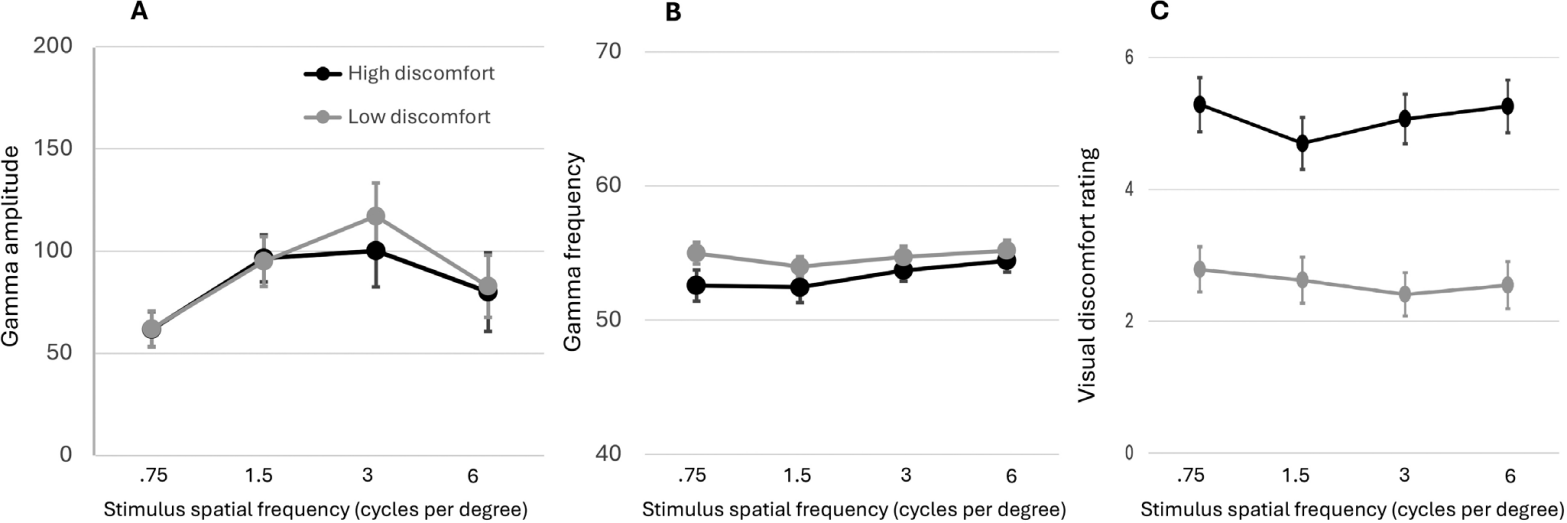
Study 2 found no difference between groups in gamma power (A) or frequency (B), despite clear difference in visual discomfort experienced to the inducing stimuli (C).

Time-frequency plots for Study 2 are displayed in Figure 4, for each spatial frequency for each group. We found no significant difference between groups in either gamma amplitude or frequency (Fig. 5A, B). There were no main effects of group (F(1,47)<0.1, ω^2^ < 0.001, F(1,47) = 1.9, ω^2^ = 0.01) or interactions of group with spatial frequency (F(3,141) = 0.6, ω^2^ < 0.001, F(3,141) = 0.8, ω^2^ < 0.001). Bayesian ANOVAs found the most likely model to contain only spatial frequency for amplitude and null for frequency (BF vs including group difference = 3 and 2.3; BF vs including interaction = 10 and 2.8). The fitted DCM parameters did not predict pattern hypersensitivity scores or discomfort during MEG scanning for any spatial frequency (all F(8,40)<2).

There were no data quality issues. Gamma frequency correlated clearly with age (mean frequency r = -0.65, 95% CI -.46 to -.79; replicated in every spatial frequency, r =-. 62, -.64, -.59, -.39, all p < 0.005, though including age as covariate did not change the main results, group main effect F = 0.1, interaction F = 0.5). DCM parameters also predicted age in all spatial frequencies (F(8,40)>3.4, p < 0.01). Gamma amplitude peaked for 3 cpd (main effect of spatial frequency, F(3,141) = 13, p < 0.001), as previously reported (Adjamian et al., 2004). Discomfort ratings during MEG recording confirmed higher discomfort in the ‘high’ group (Fig. 5C, F(1,47) = 28, p < 0.001). Discomfort ratings correlated as expected with migraine, anxiety, and VVAS (r = 0.65, r = 0.38, r = 0.56).

Given that the inverted U curve of gamma amplitude with spatial frequency appears similar to the curve of gamma amplitude with stimulus speed (Orekhova et al., 2018), we performed an exploratory test analogous to characterising ‘gamma suppression’ at high stimulus speed (Manyukhina et al., 2021; Orekhova et al., 2018, 2019). We calculated the percentage amplitude reduction between 3 and 6 cpd for each participant and compared these across groups. There was no evident difference (t(47) = 1.8, BF = 0.99).

Lastly, given that individuals in the high discomfort group differed in the stimulus that was most uncomfortable for them, we calculated the correlation between discomfort ratings and gamma amplitude or frequency within each participant, across spatial frequency. We found no consistent relationship (for amplitude, 9/22 correlations were positive, the rest negative; for frequency, 11/22 were positive).

## 4 Discussion

Contrary to our hypothesis, there was no association between any sensory sensitivity measure and visual gamma, or with the parameters of the DCM. Of particular interest, following Shaw et al. (2017), would have been inhibitory gain control on the superficial pyramidal cells (G7 in Fig. 1C) and inhibitory drive from the inhibitory interneurons (G11) but neither of these parameters showed any association with sensory sensitivity. These null results replicated across both studies, using approaches with different merits (discussed below). They could not be explained by data quality or limited variance, since all the sense-checks showed the expected behaviour of our key measures: gamma frequency with age, gamma amplitude with spatial frequency, sensory discomfort with migraine, anxiety, autism traits, and visually-induced dizziness.

It is worth remembering that the stimuli were causing discomfort for some participants while their gamma oscillations were measured. Yet the neural underpinning of this perceptual experience was in no way evident in the simultaneously induced gamma or DCM parameters. While perceptual experience should not be assumed to correspond directly with any given measurable ‘neural sensitivity’ (Ward, 2019) or ‘sensory-related neural excitability’ (He et al., 2023), we were testing a specific prediction arising from one of the main theories of aversive sensory discomfort: individual differences in excitation/inhibition in sensory cortex.

### 4.1 Sensory discomfort does not reflect excitation/inhibition in sensory cortex?

How does our result square with previous evidence supporting hyperexcitation of visual cortex? In fact, the majority of studies assessed differences between stimuli, not between individuals. Stimuli that can evoke discomfort tend to produce larger neural or hemodynamic responses, compared to more comfortable stimuli (Patterson Gentile & Aguirre, 2020; Haigh et al., 2013; Huang et al., 2011; Le et al., 2017; Lindquist et al., 2021; O’Hare et al., 2015, 2023), consistent with the strong gamma response seen for uncomfortable stripes, and consistent with inefficient coding for stimuli that deviate from natural scenes (Penacchio et al., 2023; Ward, 2019; Wilkins, 1995). In some ways, this relationship also broadly mirrors threshold sensitivity functions, as in the curve of gamma against spatial frequency (Adjamian et al., 2004) and speed (Orekhova et al., 2018), tempting us to assume a direct relationship between *individual differences* in measured sensitivity (ability to detect or discriminate stimuli) and aversive sensory discomfort (subjective sensitivity) underpinned by differences in the visual cortex.

It is intuitive that the source of individual differences might arise from well-studied stimulus-related processes, but there are cautionary examples where this is not the case (Borsboom et al., 2009; Boy & Sumner, 2014; Hedge et al., 2018, 2022). Across individuals, it turns out there is often no correlation between discomfort and objective threshold measures (He et al., 2023; Schulz & Stevenson, 2022; Ward, 2019), and neither is there a compellingly strong correlation between threshold measures themselves for different types of stimuli (Bosten et al., 2017; Mollon et al., 2017). Therefore, there is no trait of ‘objective sensitivity’ to explain the trait of subjective sensitivity (which does strongly correlate across different types of stimuli; Price et al., 2025).

Turning then to the direct evidence relating individual differences in discomfort to visual cortex activation, it is much sparser than the data relating discomfort-related stimulus properties to cortical activity. Bargary et al. (2015) found greater BOLD in visual and parietal areas for hypersensitivity participants viewing glare stimuli. Group differences between people with migraine or autistic individuals and comparison participants have been reported with BOLD, PET and ERPs (Boulloche et al., 2010; Green et al., 2013; Haigh et al., 2019; Huang et al., 2003, 2011), but not always with strong statistical power and absent group differences have also been reported (Patterson Gentile & Aguirre, 2020). A recent study found no correlation between visual discomfort and visual cortex GABA concentration measured with MR spectroscopy, convergent with our null result for gamma oscillations (Jurkovičová et al., 2024), although strong conclusions from spectroscopy are difficult (see discussion below).

It is not new that gamma responses diverge from BOLD (Hermes et al., 2017; Swettenham et al., 2013). If we accept that gamma power and frequency more directly index visual cortex excitation/inhibition balance than haemodynamic signals (further discussed below), then our null results would seem to indicate that individual differences in visual hypersensitivity are not caused by relative hyper-excitability or lower inhibition within the visual cortex. In turn, this may lend support to other theories of hypersensitivity, involving prediction, habituation, attention, and connectivity beyond sensory cortex (Green et al., 2017; Huang & Wilkins, 2021).

### 4.2 Other theories of sensory discomfort?

Beyond the excitation/inhibition theory, three other categories of explanation for sensory hypersensitivity were delineated by Ward (2019). The second major type of theory involves a collection of concepts about how sensory information is prioritised (attended to) and integrated with expectations or predictions (priors), relying on an optimal integration of feedforward and feedback signals (e.g. Pellicano & Burr, 2012). These theories have been most discussed regarding autism, rather than sensory sensitivity itself, and operate at a different level of explanation than cortical excitation/inhibition (algorithmic vs implementation; Marr, 1982). While local excitation/inhibition differences would have an effect on feedforward/feedback integration, the reverse is not necessarily the case: gamma would not necessarily change if there was a difference in feedforward/feedback, depending on the implementation. For example, lesser network habituation or adaptation to ongoing or predictable sensory signals (Pellicano et al., 2007; Puts et al., 2014) would not necessarily be revealed in sustained gamma oscillations, which do not show much adaptation after the decay of the initial onset-related broad-band response. One paper, however, appears to indicate an opposite effect: higher discomfort was associated with more adaptation (Jefferis et al., 2024).

Ward (2019) also distinguishes the idea of more intrinsic (stimulus-independent) noise from stimulus-dependent noise. Both these ideas have been related to excitation/inhibition balance, often without explicit distinction. In the cortical circuit model used here, a change in spontaneous spiking (noise) would require a change in parameters that would also affect oscillations. Therefore, if the model is an appropriate extraction, our null results do not support a theory of heightened intrinsic noise.

The fourth category of theory postulates increased propagation of sensory signals to other areas. For example, greater activation of, and connectivity between, amygdala and other limbic regions associated with sensory hyper-sensitivity in autism (Green et al., 2013, 2017). Connectivity differences are also suggested in migraine (Huang & Wilkins, 2021; Stankewitz et al., 2021) and central sensitisation for chronic pain (Cagnie et al., 2014). Our results may suggest that such connectivity differences may be the more fruitful line of enquiry for why people differ in hypersensitivity, rather than individual differences in the response of the visual cortex itself.

### 4.3 Does human visual gamma reflect excitation/inhibition?

One possible explanation for our null results would be that *individual differences* in visual gamma (and DCM model parameters) do not reflect cortical excitation/inhibition as currently understood. The association of human visual gamma with excitation/inhibition has been mainly supported through within-participant pharmacological modulations (Campbell et al., 2014; Lozano-Soldevilla et al., 2014; Magazzini et al., 2016; Saxena et al., 2013). While these, like the previous animal research (Bartos et al., 2007; Whittington et al., 2000), clearly indicate that gamma oscillations depend on GABAergic inhibition, they do not confirm the source of *individual differences* in gamma characteristics.

Individual differences in visual gamma appear robust and meaningful, as shown by the replicated correlation of frequency and age (Gaetz et al., 2012; Muthukumaraswamy et al., 2010; Orekhova et al., 2015; Robson et al., 2015) stability over time (Hoogenboom et al., 2006; Muthukumaraswamy et al., 2010) and tight correlation between identical twins (van Pelt et al., 2012). But whether these individual differences index excitation/inhibition is not quite as secure. Modeling suggests the developmental emergence of visual gamma oscillations is related to the maturation of the E/I balance (Rhodes et al., 2024), and early studies showed a correlation between gamma peak frequency and resting GABA concentration measured with MR spectroscopy in the visual cortex (Edden et al., 2009; Muthukumaraswamy et al., 2009) and other areas (Chen et al., 2014; Gaetz et al., 2011). However, follow-up study did not replicate this correlation (Cousijn et al., 2014). MR spectroscopy is inherently noisy (Mikkelsen et al., 2016) and is a measure of global concentration in the area of measurement, not of synaptic connections; it does not, for example, correlate with TMS indexes of synaptic inhibition (Stagg et al., 2011; Tremblay et al., 2013) and modeling indicates that increasing GABAergic conductance on excitatory or inhibitory cells in the circuit influences oscillation power in opposite directions (Zachariou et al., 2021).

A more direct assessment of synaptic characteristics can be made with Flumazenil-PET to measure resting-levels of GABA_A_ receptor density, although this technique is also not a direct measure of inhibition strength in specific circuit connections. Nevertheless, this technique has shown correlations with gamma frequency and amplitude (Kujala et al., 2015), albeit with small numbers.

Group studies also lend converging support. Visual gamma frequency and amplitude were lower in people with schizophrenia (Grent-‘t-Jong et al., 2016; Shaw et al., 2020), correlated with symptom severity and with a psychophysical task thought to tap local inhibition; further, DCM modelling provided a coherent account via connectivity between inhibitory interneurons and superficial pyramidal cells (Shaw et al., 2020). Interestingly MRS-measured GABA concentration did show a corresponding group difference between schizophrenia and comparison participants, but not a significant correlation with gamma, further highlighting the difficulty of interpreting MRS GABA.

Taken together, there is not unequivocal evidence that individual differences in visual gamma directly reflect excitation/inhibition balance, but the integration of animal neurophysiology, human pharmaco-modulation and DCM using a canonical microcircuit model of V1 macro-columns (Shaw et al., 2017) provides a coherent account that has been successfully applied to both group differences and individual differences. There is no other more plausible explanation at present.

### 4.4 Inhibitory differences not captured by our methods?

While we concluded above that individual differences in gamma probably do partially reflect genuine differences in excitation/inhibition, can excitation/inhibition also differ between individuals in ways that are invisible to the oscillation measurement and DCM modelling we implemented?

Another approach to stimulating and measuring gamma oscillations is to use different stimulus speeds (for similar black and white gratings as used here). Gamma frequency increases monotonically with stimulus speed, but gamma power shows an inverse U-shape, rising with static to slow speeds and falling again for fast speeds (Orekhova et al., 2018, though see Muthukumaraswamy & Singh, 2008). Most studies of gamma oscillations utilize static or slow speed stimuli because they reliably generate gamma, but interestingly, it was not peak gamma, but rather the size of the reduction at faster speeds that was found to correlate with sensory sensitivity in two studies (Manyukhina et al., 2021; Orekhova et al., 2019). As seen in our results (Fig. 5A), there is also an inverse-U curve of gamma power with spatial frequency (Adjamian et al., 2004) but we did not find that the reduction for high spatial frequencies was associated with sensory sensitivity. Therefore, a potentially different signal appears to be captured by the reduction in gamma at high stimulus speeds, which has not so far directly informed the computational models of gamma oscillations.

This reduction in gamma power for high-speed stimuli has been called gamma suppression (Orekhova et al., 2018). Rather than reflecting the number of responsive visual neurons, as is the assumed explanation for spatial frequency tuning of BOLD and gamma (Singh et al., 2000), it is suggested that there is an active suppression mechanism that counteracts growing excitation in response to intensive visual stimulation, as a mechanism of gain control (Orekhova et al., 2020). If this suppression mechanism was less effective in some individuals, it might account for visual overload (Manyukhina et al., 2021; Orekhova et al., 2019).

There is not yet a computational model that captures this gamma suppression effect. Gamma power in monkey local field potential (LFP) oscillations shows a similar shaped inverted-U shape with stimulus contrast (Hadjipapas et al., 2015; Roberts et al., 2013), and this has been modeled (Zachariou et al., 2021). In the model, increasing the external drive activating the inhibitory cells flattened the U-shape of gamma power, without changing gamma frequency (Zachariou et al., 2021, Fig. 5), producing an effect akin to the individual differences associated with sensory sensitivity in Orekhova et al. (2019). However, note that lower inhibition was associated with *more* reduction for stronger stimuli, opposite to the conceptual association of gamma suppression with less sensory hypersensitivity (but consistent with the canonical cortical circuit we used, which predicts lower gamma power with lower interneuron inhibition on superficial pyramidal cells).

Other challenges also remain: the shape of LFP gamma power is not mirrored by human MEG recordings, which show a monotonic increase in power with contrast (Hadjipapas et al., 2015; Perry et al., 2014), so models of LFP cannot be simply mapped to MEG, where longer range spatial interactions change the emergent oscillations (Hadjipapas et al., 2015).

There is a further logical puzzle. If individual differences in gamma suppression at high stimulus speeds are to be captured in the excitation/inhibition parameters of any model variant based on current models, such parameters would also influence gamma at other speeds, including the gamma paradigm we used in our study. Therefore, it is our conjecture that the correlations reported between sensory sensitivity and gamma suppression for fast speeds reflect something missing from current understanding, rather than being an index of our mainstream conception of excitation/inhibition balance in the visual cortex.

### 4.5 Strengths and limitations

We deployed different approaches in studies 1 and 2 which each have their strengths and limitations. Study 1 used a larger cohort than any previous study assessing the neural correlates of hypersensitivity, but variance and representativeness was restricted in certain ways; for example, severe migraine was screened out at recruitment. Also, for the discomfort image ratings, we used a traditional numerical scale, since been shown to be suboptimal compared to functional questions (Price et al., 2025). However, there was sufficient variance in AASP and image ratings for the expected correlations to be present between the two measures and with anxiety, AQ, VVAS, and migraine. We used a moving stimulus to increase average gamma amplitude and facilitate fixation, the attention task involved a speed change which will broaden the gamma peak because gamma frequency increases slightly with speed. We used a spatial frequency of 1.5 cpd, which may produce slightly lower gamma amplitude than 3 cpd (e.g. Figs. 4 and 5 here, and Adjamian et al., 2004 Fig. 2). However, the gamma response to 1.5 cpd was actually stronger in study 1 than the response to 3 cpd in study 2 because of the stimulus motion. Variance was sufficient in both studies to provide strong correlation with age. Study 2 had lower numbers, but recruited participants specifically with high pattern discomfort, most relevant for models of hyperexcitability of the visual cortex. We also demonstrated visual discomfort during scanning itself. While participants were requested to fixate and EOG was recorded, in neither study did the participants’ task require fixation. Future research will explore connectivity across wider brain networks and also alpha power variation.

## 5 Conclusions

Our results did not support a key prediction of the cortical hyperexcitability theory of sensory hypersensitivity. This may reflect missing understanding in our models of stimulus-induced oscillations. More likely, it points us to theories of visual hypersensitivity beyond the visual cortex, involving attention or connectivity with other systems.

## Data Availability

Data are available at: https://gin.g-node.org/CUBRIC/WAND (study 1) and https://osf.io/yrusq (study 2) Matlab code used to analyze these data to generate the virtual sensors, spectral reconstructions, and the peak gamma frequency and amplitude metrics used in this paper are available at https://osf.io/yrusq.
